# Endothelium-Dependent Vasorelaxant Effects of Dealcoholized Wine Powder of Wild Grape (*Vitis coignetiae*) in the Rat Thoracic Aorta

**DOI:** 10.1155/2016/6846084

**Published:** 2016-10-20

**Authors:** Sang Keun Ha, Ho-Young Park, Mee-Ra Ryu, Yoonsook Kim, Yongkon Park

**Affiliations:** Division of Functional Food Research, Korea Food Research Institute, Gyeonggi 463-746, Republic of Korea

## Abstract

The vasorelaxant effects of dealcoholized wild grape (*Vitis coignetiae*) wine were investigated with isolated rat thoracic aorta. In our present study, we demonstrate that wild grape wine powder (WGWP) induced relaxation of aortic rings preconstricted with norepinephrine in a dose-dependent manner (at concentrations ranging from 0.1 to 1 mg/mL). The vasorelaxant effect of WGWP was dependent on intact endothelia, which was attenuated by incubation with inhibitors of endothelium-derived relaxing factors, such as N^G^-nitro-L-arginine (nitric oxide synthase inhibitor), methylene blue (guanylate cyclase inhibitor), and indomethacin (cyclooxygenase inhibitor). Moreover, treatment with WGWP and atropine (muscarinic receptor antagonist) or diphenylhydramine (histamine receptor antagonist) significantly inhibited endothelium-dependent vasorelaxation. Our results suggest that WGWP induces relaxation in rat aortic rings in an endothelium-dependent manner. Results further indicate that this effect occurs via nitric oxide-cGMP pathway and prostacyclin-cAMP pathway through a muscarinic receptor and histamine receptor.

## 1. Introduction

Throughout the mid to early 20th century, it was believed that intake of wine was harmful for health and excessive alcohol consumption caused various ailments such as blood disorders, high blood pressure, cancer, infertility, liver disease, and brain damage. However, in the 1990s and early 21st century, some studies pointed out the apparently paradoxical relationship between high-fat/high dairy diet of the French people and the low occurrence of cardiovascular disease among them (termed the French Paradox). Investigations indicated that the low incidence of cardiovascular diseases in the French population could be attributed to the beneficial effects of red wine and its polyphenol constituents [1, 2], triggering considerable discussion and research to understand the health benefits of red wine. Multiple studies have reported that wine and its polyphenols have numerous health benefits such as antioxidant [3, 4], anticancer [5, 6], neuroprotective [7, 8], and immunomodulatory [9] properties. In fact, numerous lines of evidence strongly support the notion that only moderate wine consumption provides beneficial cardiovascular effects [1]. It is well known that several key molecules are involved in the French Paradox.

Red wine polyphenols affect the expression of adhesion molecules and inflammatory cytokines related to atherosclerosis [10] and prevent cardiovascular alteration associated with obesity [11]. Red wine also exerts beneficial cardiovascular effects via increased expression of human endothelial nitric oxide synthase [12]. Recently, scientists, mostly from Korea and Japan, have reported novel biological functions of Korean red wine and its raw material, wild grape (*Vitis coignetiae*, known as meoru in Korea), such as antigenotoxic [13] and hepatoprotective activities [14]. Moreover, rhapontigenin and resveratrol isolated from* V. coignetiae* inhibit angiogenesis in PC-3 prostate cancer cells [15] and food intake in mice, respectively [16]. Anthocyanins in* V. coignetiae* have anti-invasive activities and induce apoptosis in various human cancer cell lines [17–19]. The potential of wild grape wine as a functional food for neuroprotective, antioxidant, and antiproliferative effects has been also reported [8, 20, 21]. The* Vitis hybrid*-*Vitis coignetiae* red wine showed high antihypertensive angiotensin I-converting enzyme inhibitory activity and antioxidant activity [22]. However, the vasorelaxant properties of wild grape wine powder (WGWP) have not yet been reported. In the present study, we investigated the effects of WGWP on the development of vasorelaxation under* in vivo* conditions. We demonstrate that this effect occurs via the NO-cGMP and prostacyclin-cAMP pathways through muscarinic and histamine receptors, respectively.

## 2. Materials and Methods

### 2.1. Chemicals

All chemicals, including norepinephrine (NE), N^G^-nitro-L-arginine (L-NNA), methylene blue, indomethacin, atropine, diphenylhydramine, propranolol, and [D-Pro^2^, D-Trp^7,9^] substance P, were purchased from Sigma Chemical Co. (St. Louis, MO, USA).

### 2.2. Preparation of WGWP

Wild grape wine was produced according to the method of Kim et al. [21] using Korean wild grape (*V. coignetiae*) and stored at −20°C until used. The content of moisture in wild grape wine was 74.4 ± 0.7%. Wine was concentrated by dealcoholization and then freeze-dried to make WGWP.

### 2.3. Measurement of Polyphenol Contents in WGWP

To measure the levels of polyphenols, wine samples were injected into Amberlite XAD-2 column. Adsorbed phenolic compounds were dissolved using methanol and then concentrated under vacuum. The contents of polyphenols in wine extract were determined using high-performance liquid chromatography (HPLC, Agilent 110 system, HP Co., USA) analysis. Reverse phase chromatographic separation was carried out in a Grom-sil 120 ODS-3 C_18_ column. The experimental conditions included an isocratic binary system of 2% acetic acid and methanol. Changes in absorbance at 254, 280, and 360 nm were recorded using a UV-VIS detector.

### 2.4. Measurement of Vascular Reactivity of Aortic Rings

Male Sprague-Dawley rats (200–250 g) were sacrificed by stunning and exsanguination. The thoracic aorta was dissected free from the surrounding connective tissues and cut into rings 2-3 mm in length. The rings were then transferred to 4 mL horizontal-type muscle chambers and bathed in physiological salt solution (PSS) containing 115 mM NaCl, 5 mM KCl, 2.1 mM CaCl_2_, 1.2 mM MgSO_4_, 25 mM NaHCO_3_, 11 mM glucose, and 1.2 mM KH_2_PO_4_ at 37°C, in an atmosphere of 95% O_2_ and 5% CO_2_. The segments were then stripped of endothelium by gently rubbing with a moistened swab. Each experiment was performed on rings prepared from different rats. All studies were performed according to the Guiding Principles for the Care and Use of Laboratory Animals of the Ethics Committee of the Korea Food Research Institute.

All rings were equilibrated for 60 min under resting tension of 1 ×g and then exposed repeatedly to 72 mM KCl in PSS until the responses stabilized. Control contraction was then produced by addition of 300 nM NE. After sustained tension (60% of the maximal contraction in response to 72 mM KCl PSS in endothelium-intact rings) was obtained, WGWP solution was added to the bath solution. In experiments using inhibitors and antagonists, this was added 20 min before precontraction. N^G^-Nitro-L-arginine (L-NNA, nitric oxide synthase inhibitor, 10 *μ*M), methylene blue (guanylate cyclase inhibitor, 1.0 M), indomethacin (cyclooxygenase inhibitor, 10 *μ*M), atropine (muscarinic receptor antagonist, 1.0 *μ*M), diphenylhydramine (histamine receptor antagonist, 10 *μ*M), propranolol (bradykinin receptor antagonist, 1.0 *μ*M), and [D-Pro^2^, D-Trp^7,9^] substance P (substance P receptor antagonist, 5 *μ*M) were used. Vascular tone was measured using a force-displacement transducer (FT 03; Grass, West Warwick, RI, USA) connected to a polygraph system (RPS 212; Grass, RI, USA) and a computer analyzer (Power Laboratory 400, MacLab; AD Instruments, Castle Hill, Australia).

### 2.5. Data Analysis

All results are expressed as mean ± SEM. The number of rings obtained from different rats is represented by *n*. Relaxation is expressed in terms of the percentage decrease in maximal contraction caused by NE (300 nM). One-way ANOVA and the Student Newman-Keuls test were used for statistical analyses of differences between groups and *P* values < 0.05 were regarded as statistically significant.

## 3. Results

### 3.1. The Contents of the Polyphenol (mg/g) in WGWP

We measured the levels of minerals and total polyphenols in WGWP. As shown in Table 1, we measured the contents of various polyphenols such as (+)-catechin, caffeic acid, (−)-epicatechin, coumaric acid, ferulic acid, resveratrol, and quercetin dihydrate in WGWP. Among the different polyphenols, level of (−)-epicatechin was the highest (52.6 mg/g). Other polyphenols were present in the range of 1.26–33.3 mg/mL.

### 3.2. Endothelium-Dependent Relaxation by WGWP

**Figure 1:**
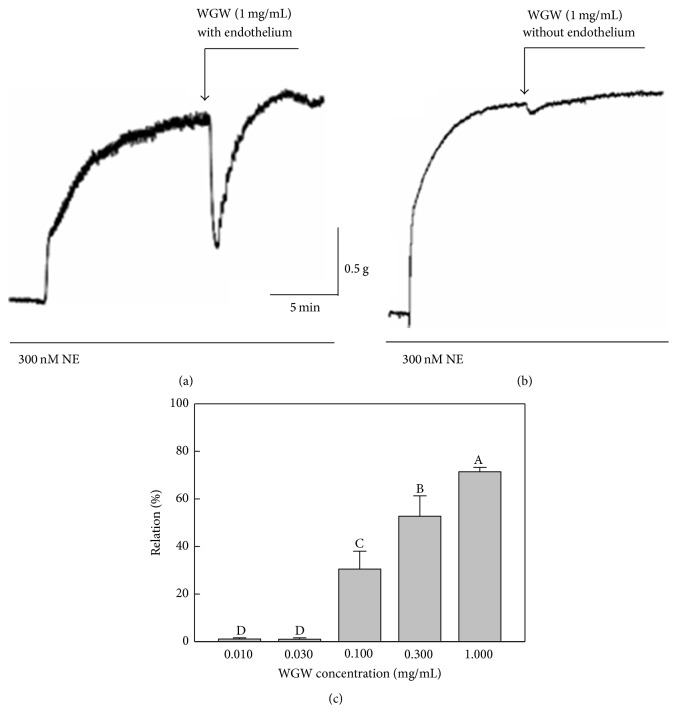
The vasorelaxant effect of WGWP in rat aortic rings preconstricted with norepinephrine. Isolated rat aortic rings without (a) or with (b, c) endothelium were preconstricted by addition of 300 nM norepinephrine (NE) and vasorelaxation was measured after treatment with WGWP ((a, b) 1 mg/mL; (c) 0.01–1.0 mg/mL). ^A–D^Means with different letters are significantly different (*P* < 0.05) by one-way analysis of variance.

To investigate the effects of WGWP on vasorelaxation, we evaluated the influence of WGWP on vasomotor tone of isolated rat thoracic aorta preconstricted with NE. As shown in Figure 1(a), muscle tension stimulated with 300 nM NE did not change upon treatment with 1.0 mg/mL WGWP in aortic rings without the endothelium. However, WGWP relaxed the endothelium-intact aortic preparation immediately by 72 ± 2% in comparison to that achieved with NE treatment only, and this effect lasted for 5 min (Figure 1(b)). As shown in Figure 1(c), WGWP also relaxed aortic rings with the endothelium in a dose-dependent manner. At concentrations of 0.1 and 0.3 mg/mL, WGWP exerted the relaxant activities by 32 ± 10 and 54 ± 9%, respectively. No effect was observed at concentrations of 0.01–0.03 mg/mL.

### 3.3. WGWP-Induced Vasorelaxation via NO-cGMP Pathway and Prostacyclin-cAMP Pathway through Muscarinic Receptor and Histamine Receptor

**Figure 2:**
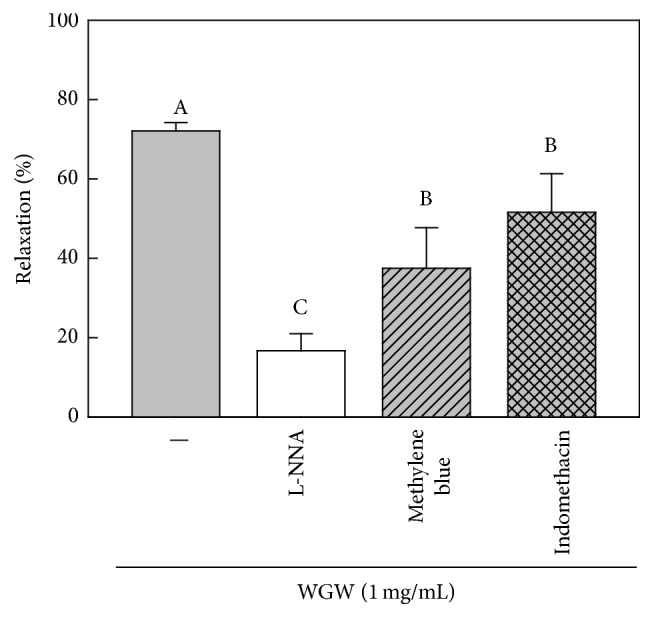
The effects of endothelium-derived relaxing factor inhibitors on WGWP-induced vasorelaxation in rat aortic rings. We pretreated endothelium-derived relaxing factors inhibitors, N^G^-nitro-L-arginine (NNA, nitric oxide synthase inhibitor, 10 *μ*M), methylene blue (guanylate cyclase inhibitor, 1.0 M), and indomethacin (cyclooxygenase inhibitor, 10 *μ*M), for 20 min and constricted rat aortic rings by 300 nM norepinephrine. Then, vasorelaxation was measured after treatment with WGWP 1.0 mg/mL. ^A–C^Means with different letters are significantly different (*P* < 0.05) by one-way analysis of variance.

To investigate the effects of WGWP on endothelium-derived relaxing factors (EDRFs), we pretreated isolated rat thoracic aorta with inhibitors, L-NNA, methylene blue, and indomethacin, followed by treatment with NE and WGWP. As shown in Figure 2, WGWP-induced vasorelaxation was attenuated by L-NNA, methylene blue, and indomethacin by 79, 50, and 29%, respectively.

**Figure 3:**
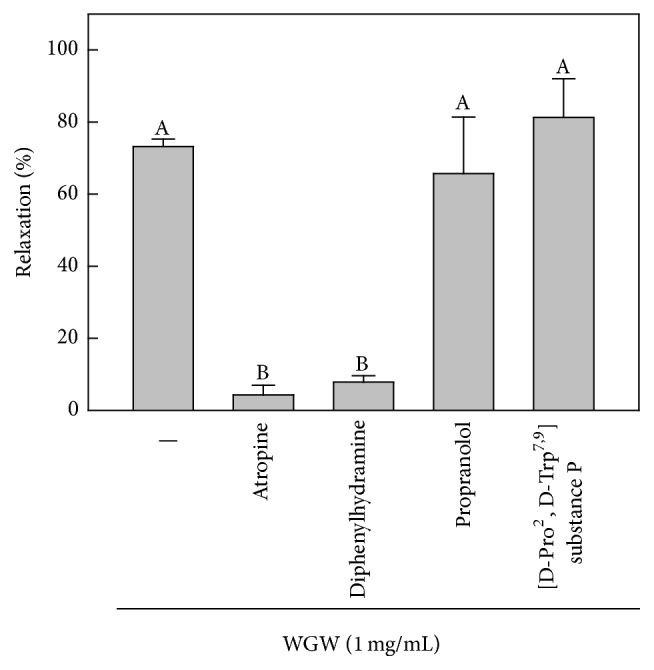
The effects of endothelial receptors antagonist on WGWP-induced vasorelaxation in rat aortic rings. We pretreated endothelial receptors antagonists, atropine (muscarinic receptor antagonist, 1.0 *μ*M), diphenylhydramine (histamin receptor antagonist, 10 *μ*M), propranolol (bradykinin receptor antagonist, 1.0 *μ*M), and [D-Pro^2^, D-Trp^7,9^] substance P (substance P receptor antagonist, 5 *μ*M), for 20 min and constricted rat aortic rings by 300 nM norepinephrine. Then, vasorelaxation was measured after treatment with WGWP 1.0 mg/mL. ^A, B^Means with different letters are significantly different (*P* < 0.05) by one-way analysis of variance.

To evaluate the influence of WGWP on the activation of endothelial receptors, we treated isolated rat aortic rings with specific receptor antagonists, atropine, diphenylhydramine, propranolol, and [D-Pro^2^, D-Trp^7,9^] substance P, and WGWP and measured vasomotor tone. As shown in Figure 3, WGWP-induced vasorelaxation was significantly attenuated by atropine and diphenylhydramine. However, propranolol and [D-Pro^2^, D-Trp^7,9^] substance P had no influence on vasomotor tone in this study.

## 4. Discussion

Many investigations have focused on the well-known relationship between wine consumption and vasorelaxation associated with cardiovascular risk. Recent studies showed that red wine consumption improves vascular function in healthy volunteers and in patients with coronary artery disease [23, 24]. In animal models, red wine and other wine derivatives (especially polyphenols) exerted endothelium-dependent vasorelaxant effects [25]. Moreover, vasorelaxant effects of not only wines, but also grape juices and grape skin extracts have been described [26]. However, the vasorelaxant properties of WGWP have not yet been reported. To investigate the vasorelaxant effects of WGWP, we evaluated the influence of WGWP on vasomotor tone of isolated rat thoracic aorta preconstricted with NE. We found that WGWP relaxed endothelium-intact rat aortic rings, significantly. However, this effect was not exerted in aortic rings with the endothelium. These results indicate that the vasorelaxant property of WGWP may depend on intact endothelia. It is well established that vasorelaxant agents such as EDRF and prostacyclin (prostaglandin I_2_) are produced and released by the endothelium, which leads to vasorelaxation of vascular smooth muscle cells and maintenance of vascular tone [27]. Nitric oxide (NO), the major EDRF, is induced by nitric oxide synthase (NOS) and leads to the enhancement of vasorelaxation through NO-cGMP pathway via guanylate cyclase [28]. In this study, the vasorelaxant effect of WGWP was attenuated by NOS and guanylate cyclase inhibitors, N^G^-nitro-L-arginine and methylene blue, significantly. Prostacyclin is important in the regulation of vasomotor tone and inhibition of smooth muscle cell growth via stimulation of cAMP production with adenylate cyclase [29]. In our present study, the cyclooxygenase inhibitor, indomethacin, also inhibited the vasorelaxation induced by WGWP in isolated rat thoracic aorta. Therefore, we suggest that the vasorelaxant activity of WGWP may be exerted mainly via the NO-cGMP pathway and partially via the prostacyclin-cAMP pathway.

Several neurotumoral mediators cause the release of EDRFs through activation of specific endothelial receptors such as muscarinic, bradykinin, substance P, and histamine receptors [30]. Therefore, to investigate the precise mechanisms underlying the vasorelaxant properties of WGWP, we treated constricted aortic rings with these receptor antagonists and WGWP and then measured vasomotor tone. Our results showed that atropine (a muscarinic receptor antagonist) and diphenylhydramine (a histamine receptor antagonist) significantly inhibited endothelium-dependent vasorelaxation induced by WGWP. Therefore, we suggest that WGWP induces endothelium-dependent relaxation in rat aortic rings via the NO-cGMP and prostacyclin-cAMP pathways through muscarinic receptor and histamine receptors.

Wines have recently received attention because they contain various bioactive constituents with health-stimulating properties. In particular, they contain a large amount of polyphenolic compounds such as flavonoids including anthocyanins and proanthocyanidins and phenolic acids [31, 32]. Many studies have shown that wine polyphenols help improve endothelium-dependent relaxation [33, 34]. From polyphenol-enriched wine, extracts induced endothelium-dependent vasorelaxation, similar to that elicited by the original wine polyphenolic extract [35, 36]. Therefore, we suggest that polyphenols of WGWP such as resveratrol contribute to the vasorelaxant properties of WGWP.

WGWP relaxed endothelium-intact aortic rings preconstricted with NE in a dose-dependent manner. Endothelium-dependent relaxation by WGWP in rat aortic rings may be exerted via the NO-cGMP and prostacyclin-cAMP pathways through muscarinic and histamine receptors. We suggest that WGWP can be used as an agent in the treatment of cardiovascular diseases associated with endothelial dysfunction.

## Figures and Tables

**Table 1:** The contents of six polyphenols (mg/g) in wild grape wine powder.

Polyphenol contents (mg/g)	
(+)-Catechin	17.3 ± 0.9
Caffeic acid	33.3 ± 1.4
(−)-Epicatechin	52.6 ± 4.7
Coumaric acid	29.1 ± 0.8
Ferulic acid	2.30 ± 0.1
Resveratrol	1.26 ± 0.1
Quercetin dihydrate	17.2 ± 0.2
